# Mesenchymal stem cells in the treatment of spinal cord injury: Mechanisms, current advances and future challenges

**DOI:** 10.3389/fimmu.2023.1141601

**Published:** 2023-02-24

**Authors:** Yuanliang Xia, Jianshu Zhu, Ruohan Yang, Hengyi Wang, Yuehong Li, Changfeng Fu

**Affiliations:** ^1^ Department of Spine Surgery, The First Hospital of Jilin University, Changchun, China; ^2^ Cancer Center, The First Hospital of Jilin University, Changchun, China

**Keywords:** spinal cord injury, mesenchymal stem cells, inflammatory factors, axon regeneration, macrophages, microglia

## Abstract

Spinal cord injury (SCI) has considerable impact on patient physical, mental, and financial health. Secondary SCI is associated with inflammation, vascular destruction, and subsequent permanent damage to the nervous system. Mesenchymal stem cells (MSCs) have anti-inflammatory properties, promoting vascular regeneration and the release neuro-nutrients, and are a promising strategy for the treatment of SCI. Preclinical studies have shown that MSCs promote sensory and motor function recovery in rats. In clinical trials, MSCs have been reported to improve the American Spinal Injury Association (ASIA) sensory and motor scores. However, the effectiveness of MSCs in treating patients with SCI remains controversial. MSCs promote tumorigenesis and ensuring the survival of MSCs in the hostile environment of SCI is challenging. In this article we examine the evidence on the pathophysiological changes occurring after SCI. We then review the underlying mechanisms of MSCs in the treatment of SCI and summarize the potential application of MSCs in clinical practice. Finally, we highlight the challenges surrounding the use of MSCs in the treatment of SCI and discuss future applications.

## 1 Introduction

Spinal cord injury (SCI) is the most serious complication of spinal injury and usually results in transient or permanent loss of sensory, motor, and autonomic nerves below the level of injury (1). The overall global incidence of SCI is 10.5 per 100,000 persons (2). Although 94% of patients with acute traumatic SCI survive treatment, survival is greatly reduced due to post-injury complications (3). Current therapeutic approaches for SCI that are part of standard care include the reduction of fractures, surgical decompression of the spinal canal and stabilization of the spine, and rehabilitation. However, standard treatment does not directly promote neuroregeneration, but may only reduce the effects of secondary injury and improve the conditions under which the endogenous mechanisms of repair act. The ideal result is to achieve rapid recovery of neurological function after the release of compression by medical and surgical intervention (4). However, there are currently no optimal treatment strategies to repair damaged nerve cells.

Regenerative medicine strategies based on cell therapy have attracted interest in recent years (5). Mesenchymal stem cells (MSCs) have the ability to differentiate and self-proliferate, and there has been an increasing focus on their use as potential therapeutic agents for a variety of diseases. MSCs release cytokines and exosomes to reduce inflammation at the site of injury. MSCs also release vascular endothelial growth factor (VEGF), nerve growth factor (NGF), glia-derived neurotrophic factor (GDNF) and brain-derived neurotrophic factor (BDNF) to promote nerve cells regeneration and inhibit glial scarring (6, 7). The use of MSCs represents a promising approach for SCI cell therapy. MSC transplantation therapy for SCI has been clinically proven to promote sensory and functional recovery in patients with SCI (8–11). However, stem cells have the ability to differentiate into multiple cell types and transplantation has been associated with tumour regeneration. Tumour regeneration is a reason why stem cell transplantation investigated with caution (12). In addition, the low survival rate of stem cells under the hostile conditions of injury is also one of the main factors limiting their clinical application (13). In this paper, we describe the microenvironment alterations of the spinal cord after injury. Second, we summarize the role of MSCs in SCI. Finally, we highlight the challenges surrounding the use of MSCs in the treatment of SCI and discuss future applications.

## 2 Pathophysiology of spinal cord injury

### 2.1 Primary spinal cord injury

Primary SCI is usually caused by trauma resulting in fracture or dislocation of the vertebrae and compression, tear, or transection of the spinal cord (14). Spinal cord compression is the most common mechanism and is often accompanied by disruption of the vasculature and damage to the blood-spinal cord barrier (BSCB) (3). In the healthy spinal cord, the BSCB is essential to maintain spinal cord homeostasis of spinal cord with stability (15). The concept that the spinal cord is privileged from normal immune surveillance is derived from the fact that in the healthy central nervous system there are no peripheral immune cells (16). The BSCB can resist foreign immune cells and toxic metabolites and maintain the stability of the spinal cord microenvironment. When the BSCB is destroyed, pro-inflammatory cytokines (TNF-α, IL-β, and IL-6), free radicals, and toxic substances can enter the area of injury, aggravating neuronal damage.

### 2.2 Secondary spinal cord injury

Secondary SCI is characterized by a series of cellular and molecular changes that begin within minutes of the primary injury. Secondary injury consists of three consecutive and overlapping phases: acute (within 48 hours of injury), subacute (48 hours to two weeks after injury), and chronic (lasting up to six months after injury) (17). Secondary injuries usually worsen the injury and cause permanent harm to the patient.

Disruption of the microvascular supply aggravates cell death a few minutes after SCI. When spinal cord cells are destroyed, damage-associated molecular patterns (DAMPs) are released, which induce a potent inflammatory response (18). With the activation of pattern recognition receptors (PRRs), resident and peripheral immune cells are recruited at the lesion site (19). Resident microglia, astrocytes, and peripheral-derived immune cells upregulate the expression of inflammatory factors TNF, IL-1, and IL-6 (20). The upregulation of inflammatory factors further aggravates the extensive infiltration of immune cells, which is the main cause of neurodegeneration (21). Neutrophils are known to be the first peripheral cells recruited at the site of injury (22). Reactive oxygen species (ROS) and matrix metalloproteinases (MMPs), released by neutrophils when they engulf necrotic matter and debris, lead to secondary tissue damage (23). Unlike other immune cells, the neutrophil response begins within one hour of injury and the increase in neutrophils persists at the site of injury for 3 days (24).

Microglia and monocytes-derived macrophages (MDMs) are the main cell types that trigger neuroinflammatory responses (25). Microglia are resident immune cells in the central immune system and have immune defence functions and maintain the stability of the nervous system (26). In the healthy spinal cord microenvironment, microglia are in a stable state. In the early stages of injury, the phagocytic ability of microglia allows removal of the surrounding necrotic tissue and debris (27). The positive effect of microglia at the site of injury lasts only one week, and subsequently, activated microglia and macrophages also release ROS and pro-inflammatory factors, leading to secondary damage (27, 28). Microglia, astrocytes, and oligodendrocytes form dense boundary structures of glial scars near the damaged tissue (29). The formation of glial scarring prevents immune cell infiltration and reduces inflammation (30). However, glial scarring is also an important barrier to neuronal regeneration after SCI (27). Microglia are macrophages residing in the central system, and after SCI, microglia are activated before bloodborne macrophages (31). Activated microglia then recruit bloodborne macrophages. Studies have shown that after SCI, M1 macrophages are mainly derived from microglia, and a small number of these are derived from blood-derived macrophages (32). In SCI, activated microglia secrete inflammatory factors such as IL-β, TNF-α, and IL-6 to promote the infiltration of blood-derived macrophages into the surrounding damaged tissue (33). Bloodborne macrophages include pro-inflammatory M1 and anti-inflammatory M2. Compared with microglia, M1 macrophages have a stronger phagocytic ability (34). In the subacute phase of SCI, M1 macrophages release IL-β, TNF-α, IL-6, interferon-γ, NO, and ROS, resulting in vascular endothelial damage and axonal damage. M2 macrophages release anti-inflammatory cytokines and neurotrophic factors (IL-4, IL-10, IL-13, transforming growth factor β (TGF-β) and insulin-like growth factor (IGF)) to provide the environment for neural regeneration (35). The SCI microenvironment is complex and changeable, and the regeneration of damaged neurons and axons cannot be achieved by self-regulation alone (36). Therefore, it is necessary to reverse the hostile SCI microenvironment to promote SCI recovery.

## 3 Mesenchymal stem cells regulate spinal cord injury

MSCs derive from a wide range of sources and have self-proliferation and multidirectional differentiation capabilities (37). MSCs are widely used in cell therapy and regenerative medicine because of their immunomodulatory and tissue repair effects (5, 38). MSCs can be isolated from almost all tissues, including bone marrow, adipose tissue, amniotic fluid, umbilical cord, liver, and heart (39). MSCs isolated from various tissues exhibit various cell surface markers that can be used for a range of treatment options. MSCs are nonimmunogenic, highly viable and are known to provide structural support in SCI (40). The advantage of MSCs is that they are easy to isolate and preserve, and do not raise specifically ethical issues (41). The most commonly used types of MSCs in clinical practice are bone marrow mesenchymal stem cells (BM-MSCs), human umbilical cord mesenchymal stem cells (HUC-MSCs), and fat-derived mesenchymal stem cells (AD-MSCs) (42).

BMSCs have strong differentiation potential under various induction conditions and can be divided into different subtypes of osteoblasts, chondrocytes, adipocytes, fibroblasts, and neurons and glial cells (43). BMSCs mainly play a role in inhibiting immunity against inflammation, promoting the transition of M1 macrophages to M2 type and releasing neurotrophic factors (7). The disadvantage of BMSCs is that they require patients to undergo bone marrow aspiration under local anaesthesia. AD-MSCs are derived from adipose tissue. A key feature of AD-MSCs is that they can be obtained in large quantities without causing extensive damage. Compared to BMSCs, AD-MSCs release growth factors, extracellular matrix molecules, and proteases to promote angiogenesis and wound healing (44). HUC-MSCs have stronger differentiation and proliferation capabilities than BMSCs (45). In addition, HUC-MSCs are small in size, pass through the BSCB system more easily, and do not cause fat embolism and vascular embolism (46). The role of MSCs in SCI can be broadly summarized as suppressing immunity against inflammation, releasing nutritional factors to promote neurological recovery, and stimulating angiogenesis to remodel BSCBs.

### 3.1 Anti-inflammatory role

**Figure 1:**
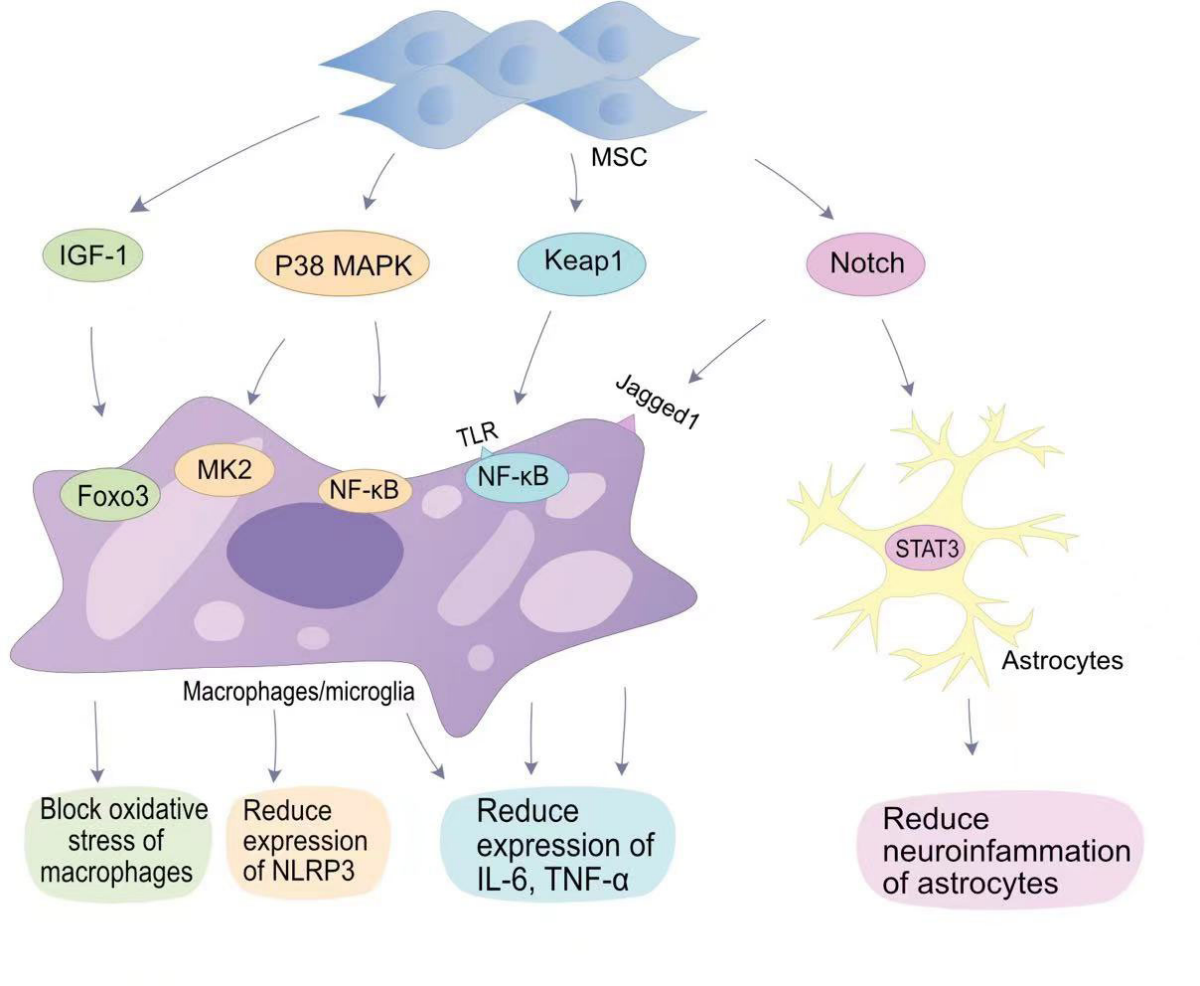
MSCs in anti-inflammatory signaling pathways in SCI. MSCs reduce oxygen partial pressure at the site of injury through the IGF-1-Foxo3 signaling pathway.MSCs reduce NLRP3 expression through the P38 MAPK-MK2 pathway and MAPK-NF-κB pathway. MSCs bind to TLR on the surface of macrophages/microglia *via* Keap1, thereby reducing IL-6 and TNF-α expression by NF-κB; MSCs reduce neuropathic inflammation through the Notch-STAT3 pathway, which is beneficial for SCI repair.

The most attractive aspect of MSCs in regulating regeneration is their unique immunomodulatory ability (47). The immunomodulatory mechanism of MSCs is mainly mediated by direct contact between cells and immune cells and paracrine activity (48). When SCI occurs, microglial activation and macrophage activation release a large number of cytokines and growth factors, including TGF-β, basic fibroblast growth factor, IL-6, andIL-1 (49). Transplantation of MSCs reduces the inflammatory response after SCI (**Figure 1**). The inhibition of inflammation by MSCs is more dependent on the realization of exosomes produced by their paracrine effect. Exosomes are membrane vesicular structures approximately 30-150 nm in diameter produced by MSCs through the paracrine pathway. As a novel intercellular communication substance, extracellular vesicles (EVs) are involved in cell proliferation and inhibit apoptosis and inflammation to mediate tissue repair (50). EVs can act as a vehicle to target inflammatory regions *in vivo*, releasing biomolecular cargoes to regulate the inflammatory response at SCI sites (51). Transplantation of BMSCs into a SCI rat contusion model significantly upregulated the number of M2 macrophages at the injury site and downregulated the number of M1 macrophages, while the levels of IL-4 and IL-13 increased, and the levels of TNF-a and IL-6 decreased (52).

The classical mitogen-activated protein kinases (MAPKs) pathway (p38 MAPK), also known as stress-associated protein kinases, can be activated during inflammation (53). In SCI, p38 MAPK-mediated inflammatory responses have been demonstrated. Toll-like receptors (TLRs) on macrophages upregulate the expression of iNOS, cyclooxygenase 2, IL-6, and TNF-α in response to a signal by p38 MAPK (54). In addition, p38 MAPK promotes the translation of cytokines in NK cells and T cells by upregulating the MAPK interaction kinase (Mnk) pathway (55). Therefore, p38 can mobilize major SCI-associated pro-inflammatory cytokines in post-traumatic inflammatory processes (53). Li et al. found that HUC-MSCs inhibit p38 activation in microglia and macrophages and reduce SCI central inflammation by inhibiting TLR4 and NF-κB signalling pathways (56). The miRNA delivered by BMSC-EVs targets TLR4 and inhibits the activation of the NF-κB pathway, alleviating the inflammatory response (57–59). Complement (C3/C5) is involved in the NF-κB signalling pathway. Zhao et al. found that BMSCs-EVs inhibit complement mRNA synthesis and release, and inhibit the activation of NF-κB signalling by binding to microglia (60). Blocking the activation of NF-κB signalling reduces the release of pro-inflammatory inflammatory factors.

The Notch pathway is a highly conserved signalling pathway that plays a role *in vivo* by promoting cell differentiation and stem cell proliferation, and maintaining cell motility (61). The Jagged1/Notch1 signalling pathway plays an important role in endogenous neurogenesis and reducing inflammation at the site of injury, and is considered to be an important target for reducing inflammation after SCI (62). Notch1 was confirmed in SCI mouse models with an initial elevation on the first day after injury which persisted until 14 days after injury. After Zhou et al. blocked the Jagged1/Notch1 signalling pathway using Jagged1 siRNA, the expression of the pro-inflammatory factors IL-1β, IL-6, and TNF-α in SCI mouse models was significantly reduced (63). MSCs transplantation can hinder Jagged1/Notch1 signalling pathway conduction after SCI, resulting in weaker expression of Notch and its downstream factors in microglia/macrophages (63). Astrocytes mainly differentiate into A1 neurotoxic-reactive astrocytes and A2 protective astrocytes in central nervous system diseases (64). The signalling mechanism that regulates astrocyte responsiveness after SCI is an important target for alleviating neuroinflammation. Notch1 can alter STAT3 phosphorylation (65). MSCs blocking the Jagged1/Notch signalling pathway can indirectly inhibit phosphorylation of the JAK/STAT3 signalling pathway in A1 neurotoxic-responsive astrocytes to achieve anti-inflammatory effects (63).

Tristetraprolin (TTP) is a substrate of p38MAPK-activated protein kinase 2 (MK2) (66). The p38/MK2 pathway is a key regulator of TTP expression, stability, and function. Previous studies have shown that MK2 can promote the expression of nucleotide-binding domain-like receptor protein 3 (NLRP3) inflammosomes by activating TTP, thereby promoting the inflammatory response (67). Li et al. showed that HUC- MSCs can reduce the production of NLRP3 inflammasomes by inhibiting the MK2/TTP signalling pathway (68). At the same time, MSCs can reduce spinal neuronal apoptosis by suppressing the expression levels of pro-caspase-1 by inhibiting the MK2/TTP signalling pathway. Mohamadi et al. also demonstrated that MSCs transplantation can reduce the expression of NLRP1 inflammasome components (NLRP1, and caspase-1) and pro-inflammatory factors (IL-1β, IL-18 and TNF-α) (69).. Furthermore, Huang et al. confirmed that fat-derived MSCs exosomes can target the elimination of NLRP3 at the SCI site and reduce inflammatory cytokine production (70). The reduction of peri-SCI inflammation by MSCs-EVs has been widely demonstrated (51).

The nuclear factor erythroid 2 (NFE2)-related factor 2 (Nrf2) signalling pathway is involved in antioxidative stress. Nrf2 can counteract the NF-κB-driven inflammatory response, endoplasmic reticulum stress and autophagy damage, and is an important factor in oxidative stress in the body (71). Keap1 is an endogenous inhibitor of Nrf2, and under basal conditions, Nrf2 is degraded by cytoplasmic Keap1 chelation combined with targeted proteasomes (72). MSCs are able to block the expression of Keap1 and reduce oxidative stress caused by SCI. miR-200a is a small interfering RNA, miR-200a targets the Keap1 3′-untranslated region (3′-UTR), resulting in degradation of the mRNA encoding Keap1 (73). Wang et al. found that the miR-200a released by MSCs can target Keap1 and inhibit the expression of Keap1, thereby improving the level of oxidative stress in SCI (74).

### 3.2 Promotion of axon regeneration

**Figure 2:**
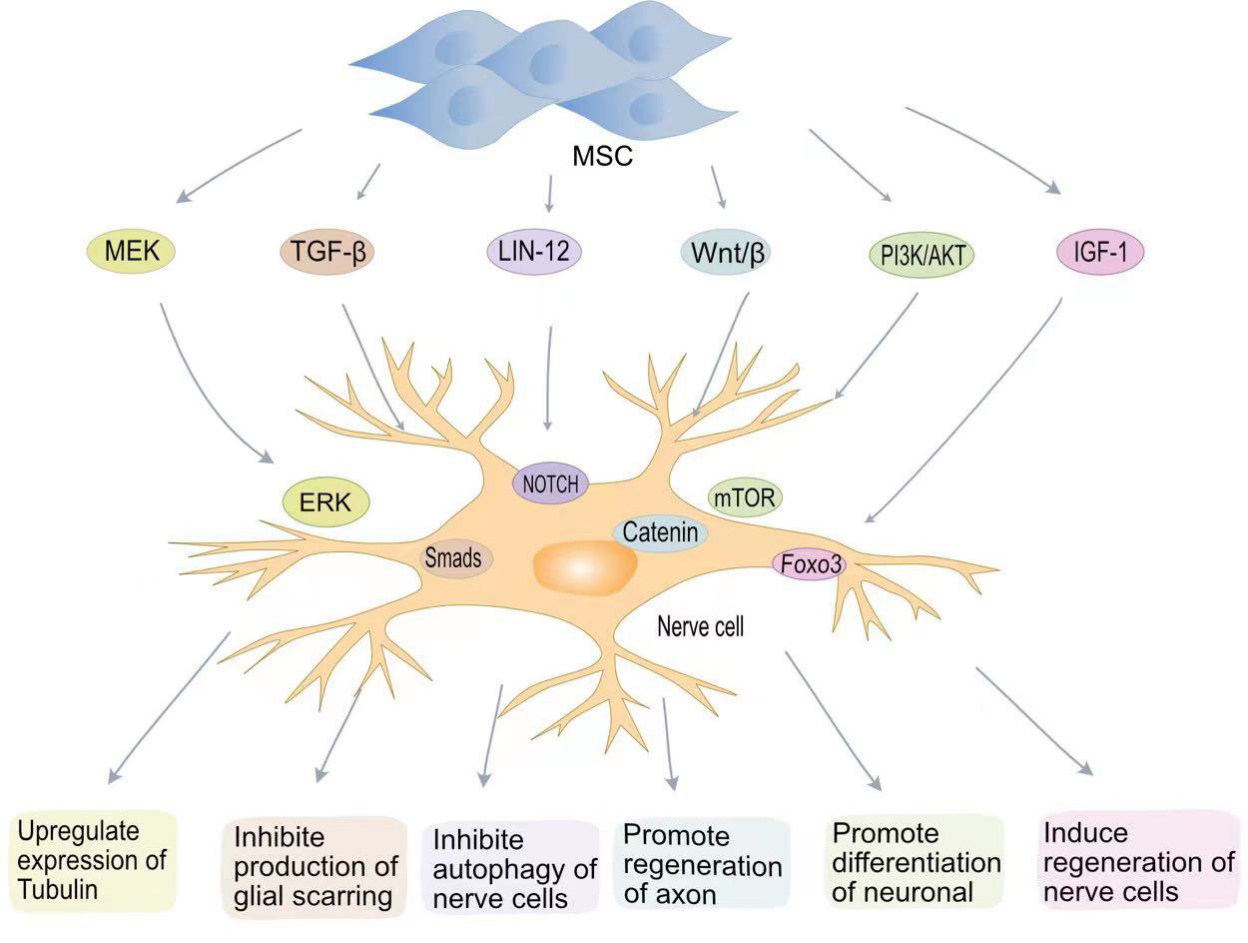
MSCs promote nerve regenerative signaling pathways in SCI. MSCs promote tubulin expression through the MEK-EPR signaling pathway; MSCs inhibit TGF-β to produce glial scarring; MSCs inhibit neurocyte autophagy *via* the Lin12-Notch pathway; MSCs promote axon regeneration through Wnt/β-catenin; MSCs induce neuronal cell differentiation *via* the PI3K-mTOR pathway. MSCs induce neural regeneration *via* the IGF-1-Foxo3 signaling pathway.

The goal of treatment after SCI is to repair the damaged nerve cells and restore patient nerve function. Neuronal destruction and loss of nerve cells after SCI are major factors in neurological dysfunction. Although the differentiation of MSCs into neurons *in vivo* remains controversial, BMSCs secrete brain-derived neurotrophic factor (BDNF) and β nerve growth factor (β-NGF) to promote neuronal survival and axon regeneration (**Figure 2**) (75, 76). Studies have found that oligodendrocytes are missing with demyelination and irreversible damage to the central nervous system (77). Muniswami et al. observed that MSCs spontaneously expressed neuromarkers at SCI sites, including β III tubulin, enolase 2, and microtubule associated protein 1b (MAP1b) (78). β III tubulin is the main component of neuronal microtubules and plays a key role in axon orientation, maturation, and maintenance (79). At 14 days after SCI, an increase in the number of viable neurons and oligodendrocytes was detected. Park et al. genetically engineered MSCs to express oligodendrocyte lineage transcription factor 2 (Olig2) (80). Transplantation of engineered MSCs to the site of injury one week after SCI in a rat model allowed recovery of rat neurological function. Zhou et al. confirmed that MSC-EVs can be internalized by neuronal cells, activate MEK/ERK pathway signalling, and enhance tubulin expression (81).

The LIN-12/NOTCH signalling pathway is a key pathway controlling the proliferation and differentiation of neural stem cells (82). Autophagy is a conserved cellular process in cellular evolution that allows the degradation of damaged cytoplastic materials and organelles. After nerve cells damage, LIN-12 restricts nerve axon regeneration through autophagy (83, 84). Chen et al. found that MSCs can inhibit the NOTCH signalling pathway and promote the differentiation of neurons (85). The Wnt/β-catenin pathway can also promote neuronal differentiation (86). The Wnt family is a class of glycoproteins widely involved in neurodevelopment, axon guidance, cell proliferation, and nerve cells survival (87). Previous studies have shown that activation of the Wnt/β-catenin signalling pathway plays a key role in functional recovery and axon regeneration after SCI (88). Yin et al. found that MSCS transplantation can enhance axon regeneration after SCI by enhancing the expression of Wnt3a protein (89).

Phosphatidylinositol 3-kinase (PI3K) is a lipid kinase involved in various cellular functions, including cell proliferation, growth, differentiation, migration, and survival (90). The mTOR protein encoded by the mammalian target of rapamycin (mTOR) gene belongs to the family of serine-threonine kinases that control cellular responses to stressors such as growth factors, nutrient deprivation, and DNA damage (91). The PI3K/AKT/mTOR signalling pathway is one of the main pathways by which mammals regulate growth (91). The PI3K/AKT/mTOR signalling pathway is associated with cancer development (92). *In vitro* experiments have confirmed that blocking the PI3K/AKT/mTOR pathway aggravates neuronal damage (36). BMSCs transplantation effectively restores motor function after SCI by activating the PI3K/AKT/mTOR pathway (93). Fan et al. developed a hydrogel loaded with BMSCs-exosomes (36). The miRNA contained in BMSC-exosomes significantly reduces mRNA expression levels in iNOS, IL-6 and TNF-α and promotes macrophage M2 polarization through the NF-κB pathway. BMSC-exosomes also promote neuronal differentiation and axon regeneration by activating the PI3K/AKT/mTOR pathway.

Insulin-like growth factor 1 (IGF-1) is a protein hormone that is an important growth factor involved in the development of the central nervous system and in promoting recovery after injury or pathological processes (94). BMSCs can secrete IGF-1, and the increase in IGF-1 can maintain the BMSC survival. IGF-1 suppresses oxidative stress at the SCI site, decreasing harmful substances such as ROS. Studies have shown that IGF-1 can actively target ROS around the injury site, blocking cellular oxidative stress processes by inhibiting the transcription factor Foxo3 (95). IGF plays an important role in nerve cells regeneration. IGF-1 binds to IGF-1 receptors on neural stem cells to promote neural stem cell recruitment and differentiation (96, 97).

The presence of glial scars can resist the invasion of inflammatory factors to a certain extent, reducing the inflammatory response after SCI. However, the presence of glial scars also hinders axon regeneration and is detrimental to nervous system recovery (98). Astrocytes are the most abundant glial cells in the central nervous system and play an important role in regulating blood flow, maintaining integrity, and maintaining neuronal homeostasis (99). After SCI, activated microglia transform astrocytes into a neurotoxic state. Chondroitin sulphate proteoglycans (CSPGs) released by astrocytes are strongly associated with glial scar formation (100). Due to the presence of glial scars, the regenerated axons cannot penetrate the colloidal scar structure and therefore cannot build neural networks. MSC-EVs inhibit the activation of astrocytes and reduce apoptosis in neuronal cells (101). MSCs transplanted into SCI rats inhibited the formation of glial scarring and also changed the morphology of astrocytes, providing a supportive microenvironment for axon regeneration and functional recovery (102). MSCs can also inhibit glial scarring by lowering cytokine levels. Studies have shown that TGF-β can mediate glial scarring by activating Smads signalling in astrocytes (103). Lev et al. showed that BMSCs can reduce glial scarring and promote axon regeneration by inhibiting the TGF-β/Smads signalling pathway (104).

### 3.3 Promotion of vascular repair

Traumatic SCI usually results in the direct destruction of the blood vessels around the spinal cord. Destruction of the blood vessels results in ischemic necrosis, and secondary injury leads to cascade enlargement (105). Vascular recovery contributes to the recovery of motor function in patients with SCI, so promoting SCI vascular recovery is a new target for the treatment of SCI (106). Menezes et al. demonstrated that fat-derived MSCs secrete a large number of angiogenic factors such as vascular endothelial growth factor (VEGF), fibroblast growth factor (FGF), platelet-derived growth factor (PDGF), and IGF-1 (107). These angiogenic factors can promote pericyte recruitment, which is considered a critical step in vascular maturation (108). The migration of MSCs to the SCI site also induces endogenous cell differentiation into pericyte, promoting new angiogenesis (108). Zhang et al. confirmed that post-SCI vascular endothelial cells can absorb MSC-EVs, while normal vascular endothelial cells cannot (109). Although the underlying mechanism is remains unclear, necrotic vascular endothelial cells may be in a specific state conducive to receiving MSC-EVs. The authors reported that 57% of the vascular endothelial cells around the mouse SCI showed a vascular regeneration effect after receiving MSC-EVs and an extensive vascular network was formed around the injury over 28 days (109). Recovery of the vascular endothelium also engulfs myelin debris and inhibits fibrotic scar formation (110).

MMP released after SCI damages the BSCB by degrading the extracellular matrix (ECM) (111). MMP causes destruction of the BSCB, and penetration of inflammatory factors and neurotoxic substances aggravates secondary damage (112). Although peripheral astrocytes can rely on the glucose-1 transporter (Glut-1) to cause new angiogenesis, this weak vascular regeneration is not sufficient to reconstruct the damaged vascular system (112). Wang et al. confirmed that MSC-EVs can maintain the integrity of the BSCB by inhibiting the expression of MMP and promoting the expression of tight junction proteins and adhesion junctions (18). Cao et al. used urine-derived MSC-EVs as a vehicle to deliver angiogenesis-related proteins to the SCI site (113). EVs produced by MSCs through the paracrine pathway have been shown to promote post-SCI angiogenesis and SCI recovery.

In summary, MSCs mainly repair SCI through anti-inflammatory effects and by promoting nerve axon regeneration and vascular regeneration. MSCs promote SCI recovery by modulating multiple pathways. Although the mechanism underlying the effect of MSCs is still not fully understood, the role of MSCs has been demonstrated.

## 4 Clinical application of MSCs in the treatment of SCI

MSCs have been shown to promote SCI recovery in clinical trials. The effectiveness of MSCs on SCI recovery is influenced by a variety of conditions: mode of transplantation, dose and frequency of MSCs, timing of SCI, and type of SCI (114). At present, the commonly used methods of MSC transplantation are subarachnoid space transplantation, intravenous injection, and local injection into the injured area (115). Intravenous MSCs are prone to pulmonary embolism, and intrathecal MSCs require larger doses because the arachnoid membrane adsorbs a large number of stem cells, which is not conducive to stem cell migration (116). Transplantation (intramedullary injection) can deliver MSCs directly to the site of injury but there is a risk of increased tissue pressure and damage to the normal spinal cord. Most clinical studies have reported delivery of MSCs *via* intrathecal injection and orthotopic injection (117). Yoon et al. reported that transplantation in the acute and subacute phases could improve neurological function in 33.3% patients with SCI improved from American Spinal Injury Association (ASIA) Impairment Scale score (AIS score) A to B or C), while transplantation in the chronic phase (5.2% of patients improved from AIS score A to B or C) did not result in a significant improvement of the outcomes (118). This may be due to the fact that during the chronic phase of SCI, the formation of glial scarring acts as a physical barrier that interferes with axon regeneration (116). Although Yoon et al. demonstrated that MSC transplantation in the acute phase can promote the recovery of extension function in patients with SCI, transplantation of MSCs in the acute phase creates a cytotoxic environment for implanted stem cells due to the presence of ROS, excitatory transmitters, and inflammatory molecules (119). Transplantation in the subacute phase of SCI appears to be the optimal time for transplantation (120). In the subacute phase, the level of inflammatory factors at the SCI site is lower than that in the acute phase and glial scar formation has not yet occurred. Dai et al. injected autologous BMSCs into a chronic SCI site (121). After 6 months, the ASIA sensory and motor scores were tested, and improvement in ASIA sensory (5.4 ± 8.22) and motor scores (0.9 ± 1.07) was observed in 10 of the 20 patients who underwent MSC transplantation. There was no significant improvement in ASIA sensory (0.25 ± 0.44) and motor scores (0.10 ± 0.31) in the control group. However, this improvement in neurological function may be explained by the large number of stem cells transplanted (122–124). In the current study, the dosage of stem cells transplanted was mainly between 1×10^6^-5×10^8^ cells/kg (41).

The promotion of sensory, motor, and neurological recovery by MSCs following SCI has been widely demonstrated (**Table 1**) (125–131). Evidence indicates that treatment with MSCs did not cause significant adverse effects (134). Cofano et al. reviewed the clinical treatment of bone marrow mesenchymal stem cells and found no significant adverse effects of stem cell therapy (41). Clinical studies have reported the development of mild fever, gastrointestinal dysfunction, headache, and urinary tract infection in only small numbers of patients after MSCs transplantation (9, 120). Nirmeen et al. reported that of 43 patients with SCI undergoing MSC transplantation, 24 patients developed neuropathic pain within 3 days of transplantation (123). This finding may be related to the frequency of MSC transplantation as the 43 patients received MSCs once a month, while in other studies most patients underwent MSC transplantation only once. There have been no reports on the relationship between MSCs transplantation frequency and adverse effects. Although MSC transplantation appears to improve function in patients with SCI, the effects are not yet clear. Karamouzian et al. reported that only 45.5% of patients in the subacute phase after SCI showed improvement in neurological function after autologous BMC transplantation (120). Park et al. reported that only 3 out of 10 patients with SCI experienced improvements in daily functioning following MSC transplantation (132). Vaquero et al. reported that 44.4% of patients experienced improvement in infralesional muscle reinnervation following MSC administration (133). At present, the effective rate of MSCs in the treatment of SCI does not support the widespread use of MSCs in clinical treatment. Therefore, further research is needed to optimize the effectiveness of MSC treatment. There are many ongoing clinical trials on the use of MSCs for the treatment of SCI (**Table 2**). In these clinical trials, most MSCs were transplanted at doses of 1×106/kg or 10-100 million per dose. The types of MSCs used in registered clinical trials continued to be BMSCs, AD-MSCs and UC-MSCs. Outcomes observed included ASIA sensory and motor scores, adverse events, and neurologically associated sensory and motor movements. Some trials are at the stage of patient recruitment while others are completed and the publication of data is currently awaited. It is anticipated that these results will provide guidance on the safety and efficacy of MSCs in the treatment of SCI.

**Table 1:** Clinical application of mesenchymal stem cells in the treatment of patients with spinal cord injury.

Author year	Type of study	Time of injury	Number of patients	Number of control group	Cell transplantation methods	Stem cell type	Number of stem cells	Result
Sergiu Albu 2021 (11)	Randomized controlled study	Chronic SCI	10	10	Subarachnoid space	BMSCs	1×10^6^	In patients with chronic complete SCI, a single intrathecal infusion of MSCs results in improved sensation in segments near the site of injury.
Yoon 2007 (118)	Non-randomized controlled study	17 cases of acute SCI, 6 cases of subacute SCI, and 12 cases of chronic SCI	35	13	*In situ* transplantation	BMSCs	2 × 10^8^	The AIS grade increased in 30.4% of the acute and subacute treated patients (AIS A to B or C), whereas no significant improvement was observed in the chronic treatment group.
Saeid 2014 (120)	Cohort study	Subacute SCI	31	20	Subarachnoid space	BMSCs	1.2×10^6^	45.5% of patients reported significant recovery (from Asian A to Asian C), compared with 15% of patients in the control group showed significant recovery.
Dai 2013 (121)	Cohort study	Chronic SCI	40	20	Subarachnoid space	BMSCs	8×10^5^	Improvements in ASIA sensation (5.4 ± 8.22) and motor score (0.9 ± 1.07) were observed in 10 of 20 patients.
Nirmeen 2017 (123)	Case-Control Study	Chronic SCI	63	20	Subarachnoid space	BMSCs	5×10^6^	BMSCs improved ASIA sensory and motor scores 6 months after transplantation.
Honmou 2021 (125)	Case series	Chronic SCI	13	/	Intravenous injection	BMSCs	1×10^8^	At 6 months after MSC infusion, neurological improvement based on ASIA grade occurred in 12 of 13 patients.
Satti 2016 (126)	Case series	6 cases of chronic SCI 3 cases were subacute	9	/	Subarachnoid space	BMSCs	1.2×10^6^	From the date of transplantation, patients were only 33 months without observing adverse effects.
Cheng 2014 (127)	Cohort study	Chronic SCI	34	10	*In situ* transplantation	HUC-MSCs	4 × 10^7^	Seven of the 10 patients in the HUCMSCs group showed significant and stable improvements in exercise, self-care ability and muscle tone.
Hur 2016 (128)	Case series	Chronic SCI	14	/	Subarachnoid space	AD-MSCs	9×10^7^	ASIA motor scores were improved in 5 patients, ASIA sensory score recovery was seen in 10, ADMSCs was free of serious adverse events.
Jeong Chan Ra 2011 (129)	Case series	Chronic SCI	8	/	Intravenous injection	AD-MSCs	4×10^8^	During the 3-month follow-up, none of the patients experienced any serious adverse events associated with hAdMSC transplantation.
Yao 2013 (130)	Case series	Chronic SCI	25	/	Subarachnoid space	HUC-MSCs	1×10^7^	Patients (16%) with advanced traumatic SCI with HUC-MSCs had improved ASIA sensory and motor scores.
Zhu 2016 (131)	Case series	Chronic SCI	28	/	Subarachnoid space	HUC-MSCs	1.6×10^6^-6.4×10^6^	UCB-MNC transplantation can lead to significant recovery of movement, bowel and bladder in patients with chronic complete SCI.
Park 2012 (132)	Cohort study, single-arm study	Chronic SCI	10	/	*In situ* transplantation	BMSCs	8×10^6^	Six of the 10 patients showed improved upper extremity motor power at 6 months of follow-up.
Vaquero 2018 (133)	Cohort study, single-arm study	Chronic SCI	20	/	Subarachnoid space	BMSCs	1×10^8^	55.5% of patients experienced improvement in somatic sensation or motor evoked potentials

**Table 2:** Ongoing trials about MSCs in SCI.

Gov Identifier		Subject	Cell type	Dosage	Transplanting Methods	Phase(s)	Primary Outcome Measures			Secondary Outcome Measures		Recruitment status
NCT03505034		Intrathecal Transplantation of UC-MSC in Patients with Late Stage of Chronic Spinal Cord Injury	UC-MSC	1×10^6^/kg, once a month for 4 months	Intrathecal Transplantation	II	ASIA sensory and motor scores			IANR-SCIRFS, Electromyogram test, Residual urine		Unknown
NCT01694927		Autologous Mesenchymal Stem Cells in Spinal Cord Injury (SCI) Patients	/	/	*In situ* transplantation	II	Number of related adverse events			Functional improvement in muscle strength, Functional Improvement in sphincters control		Unknown
NCT02574572		Autologous Mesenchymal Stem Cells Transplantation in Cervical Chronic and Complete Spinal Cord Injury	BMSCs	/	*In situ* transplantation	I	Number of related adverse events			ASIA grade, AIS scores, sensorial mapping and neuropathic pain		Unknown
NCT03521336		Intrathecal Transplantation of UC-MSC in Patients with Sub-Acute Spinal Cord Injury	UC-MSC	1×10^6^/kg, once a month for 4 months	Intrathecal Transplantation	II	ASIA Score Scale,			IANR-SCIRFS, electromyogram test, residual urine		Unknown
NCT03521323		Intrathecal Transplantation of UC-MSC in Patients with Early Stage of Chronic Spinal Cord Injury	UC-MSC	1×10^6^/kg, once a month for 4 months	Intrathecal Transplantation	II	ASIA Score Scale,			IANR-SCIRFS, electromyogram test, residual urine		Unknown
NCT02574585		Autologous Mesenchymal Stem Cells Transplantation in Thoracolumbar Chronic and Complete Spinal Cord Injury Spinal Cord Injury	BMSCs	Two percutaneous injections with a 3-month interval between the injections.	Percutaneous injections	II	Number of related adverse events			ASIA grade, AIS scores, sensorial mapping and neuropathic pain		Not yet recruiting
NCT01446640		Mesenchymal Stem Cells Transplantation to Patients with Spinal Cord Injury (MSC)	BMSCs	Intravenous administration 1×10^6^/kg; intrathecal administration 1×10^6^/kg	Intravenous combined with intrathecal administration	I, II	Number of adverse events			Electromyogram and Electroneurophysiologic test, Muscle strength assessment, Motor and sensory assessment		Recruiting
NCT05671796		Autologous Marrow Stem Cell Transplantation in Patients with Subacute Spinal Cord Injury	BMSCs	50,000,000	Intrathecal transplantation	II	ASIA scores			quality of life, neuropathic pain, sensory impairment		Not yet recruiting
NCT02688049		NeuroRegen Scaffold™ Combined With Stem Cells for Chronic Spinal Cord Injury Repair	mesenchymal stem cells, neural stem cells	10 million	receive NeuroRegen Scaffold stem cells transplantation	I, II	ASIA Impairment Scale, SSEP, MEP			Independence Measures, transplantation site, Urinary and Bowel Function, Adverse Events		Enrolling by invitation
NCT04520373		Autologous Adipose Derived Mesenchymal Stem Cells for Spinal Cord Injury Patients.	ADSCs	a single dose	intrathecal delivery	II	ASIA sensory and motor scores			SSEP, NBSS, Incidence of abnormal CSF composition, adverse events		Recruiting
NCT05152290		Safety of Cultured Allogeneic Adult Umbilical Cord Derived Mesenchymal Stem Cells for SCI	UC-MSCs	100 million cells	*In situ* transplantation	I	Adverse events			ASIA scores		Recruiting
NCT01393977		Difference Between Rehabilitation Therapy and Stem Cells Transplantation in Patients With Spinal Cord Injury in China	UC-MSC	/	*In situ* transplantation	II	Electromyogram and Electroneurophysiologic test			Electromyogram and Electroneurophysiologic test		Unknown
NCT02352077		NeuroRegen Scaffold™ With Stem Cells for Chronic Spinal Cord Injury Repair	BMMCs	/	NeuroRegen scaffold with BMMCs or MSCs transplantation	I	Number of adverse events			SSEP, MEP, AIS Scale, Independence Measures,Quality of Life, VAS, bladder pressure monitory, MRI		Enrolling by invitation
NCT02917291		Safety and Preliminary Efficacy of FAB117-HC in Patients with Acute Traumatic Spinal Cord Injury (SPINE)	ADSCs	20 or 40 million cells	*In situ* transplantation	I	Number of adverse events			ISNCSCI scale, SCIM III, SSEP, MEP		Recruiting
NCT04213131		Efficacy and Safety of hUC-MSCs and hUCB-MSCs in the Treatment of Chronic Spinal Cord Injury	UCB-MSCs	100,000 cells/μL) were injected *via* 4 points,16 μL hUC-MSCs/point.	*In situ* transplantation	Not Applicable	Neurologic function score			WISCI, SCIM, KLS, MAS		Recruiting
NCT02481440		Repeated Subarachnoid Administrations of hUC-MSCs in Treating SCI	UC-MSCs	1×10^6^/kg, once a month for 4 months	Subarachnoid space	I/II	ASIA sensory and motor scores, IANR-SCIFRS			NBD, Residual urine output		Completed
NCT02152657		Evaluation of Autologous Mesenchymal Stem Cell Transplantation in Chronic Spinal Cord Injury: a Pilot Study	/	/	Percutaneous injection	Not Applicable	Adverse events			ASIA sensory and motor scores, Urodynamics		Completed
NCT05018793		Safety of Cultured Autologous Adult Adipose Derived Mesenchymal Stem Cell Intrathecal Injection for SCI	AD-MSCs	100 million cells	Intrathecal injection	I	Adverse events			ASIA sensory and motor scores		Suspended
NCT01162915		Transfer of Bone Marrow Derived Stem Cells for the Treatment of Spinal Cord Injury	BMSCs	/	Intrathecal injection	I	Adverse events			/		Suspended
NCT02981576		Safety and Effectiveness of BM-MSC vs AT-MSC in the Treatment of SCI Patients	BMSCs, AD-MSCs	3次	Intrathecal injection	I/II	ASIA scores, Adverse events			Adverse events		Completed
NCT02570932		Administration of Expanded Autologous Adult Bone Marrow Mesenchymal Cells in Established Chronic Spinal Cord Injuries	BMSCs	1×10^6^/kg once every three months, for a total of 3 times	Intrathecal injection	II	IANR-SCIFRS			Adverse events, NBDS		Completed
NCT01769872		Safety and Effect of Adipose Tissue Derived Mesenchymal Stem Cell Implantation in Patients With Spinal Cord Injury	AD-MSCs	2×10^8^	Intrathecal injection	I/II	ASIA scores			Adverse events, SSEP		Completed
NCT01274975		Autologous Adipose Derived MSCs Transplantation in Patient With Spinal Cord Injury	AD-MSCs	4×10^8^	Intrathecal injection	I	Security			/		Completed
NCT03308565		Adipose Stem Cells for Traumatic Spinal Cord Injury	AD-MSCs	100 million cells	Intrathecal injection	I	Number of related adverse events			SSEP, ASIA scores		Completed
NCT01624779		Intrathecal Transplantation Of Autologous Adipose Tissue Derived MSC in the Patients With Spinal Cord Injury	AD-MSCs	9×10^7^ once a month, three times a month	Intrathecal injection	I	MRI			Changes in neurological function, neuroelectrophysiological changes, ASIA scores		Completed
NCT04288934		Treatment of Spinal Cord Injuries With (AutoBM-MSCs)vs (WJ-MSCs)	BMSCs	/	/	I	ASIA scores IANR-SCIRFS, SCIM III			MRI		Completed
NCT01873547		Different Efficacy Between Rehabilitation Therapy and Stem Cells Transplantation in Patients With SCI in China (SCI-III)	UC-MSC	/	Intrathecal injection	III	IANR-SCIRFS			BI,MPQ, SSEP		Completed
NCT01325103		Autologous Bone Marrow Stem Cell Transplantation in Patients With Spinal Cord Injury	BMSCs	/	Intrathecal injection	Not Applicable	Security and feasibility			Functional improvement in muscle strength, Improvement of sphincters control		Completed
NCT01909154		Safety Study of Local Administration of Autologous Bone Marrow Stromal Cells in Chronic Paraplegia	BMSCs	100×10^6^, Three months later 100×10^6^	Intrathecal injection	I	Number of related adverse events			IANR-SCIRFS, SSEP,ASIA scores, Urodynamics		Completed
NCT00816803		Cell Transplant in Spinal Cord Injury Patients	BMSCs	/	/	I/II	Security			ASIA scores and MRI		Completed
NCT02165904		Subarachnoid Administrations of Adults Autologous Mesenchymal Stromal Cells in SCI	BMSCs	Once every three months, three times in total	Intrathecal injection	I	ASIA scores, IANR-SCIRFS, Barthel, SSEP, Urodynamics, MRI			Number of related adverse events		Completed
NCT02482194		Autologous Mesenchymal Stem Cells Transplantation for Spinal Cord Injury- A Phase I Clinical Study	BMSCs	/	Intrathecal injection	I	Number of related adverse events			ASIA scores, Muscle strength		Completed

UC-MSC, Umbilical Cord Mesenchymal Stem Cells.

IANR-SCIRFS, International Association of Neural Restoration Spinal Cord Injury Functional Rating Scale.

ASIA, American Spinal Injury Association.

AIS scores, ASIA Impairment Scale.

SSEP, Somatosensory Evoked Potentials.

MEP, Motor Evoked Potentials.

NBSS, Neurogenic Bladder Symptom Score.

VAS, Visual analog scale.

ISNCSCI scale, International Standards for Neurological Classification of SCI scale.

SCIM, Spinal Cord Independence Measure.

hUCB-MSCs, human umbilical cord blood-derived mesenchymal stem cells.

WISCI, Walking Index of Spinal Cord Injury.

KLS, Kunming Locomotion Scale.

MAS, Modified Ashworth Scale.

MSCs have the potential for multi-lineage differentiation, easy isolation and preservation, and rapid proliferation and homing to lesions, which have been applied to the treatment of SCI (41). MSCs from different sources have different intrinsic properties. The use of BMSCs, HUC-MSCs, and AD-MSCs has been reported in the treatment of SCI (**Table 1**). A meta-analysis by Chen et al. showed that autologous BMSCs transplantation significantly improved ASIA motor [MD = 8.01, 95% CI (4.27, 11.76)] and sensory score [MD = 17.98, 95% CI (10.04, 25.91)] (135). Liu et al. analysed 12 studies up to January 30, 2021 with a total of 642 patients and compared BMSCs with UC-MSCs for the treatment of SCI (136). Compared to UC-MSCs, BMSCs were associated with greater improvement in ASIA exercise scores (weighted mean difference [WMD], 6.67; 95% CI, 0.83–12.73) and ASIA sensory scores (WMD, 12.41; 95% CI, 3.42–21.72). This finding may be explained by the greater ability of BMSCs to secrete neuronal phenotypic markers to induce axon regeneration compared to MSCs from other sources (137).

## 5 Challenges in the clinical application of MSC therapy

MSCs have immunomodulatory abilities and play an important role in the regulation of immune responses and the development of many diseases. Studies have shown that mesenchymal stem cells are involved in the initiation, progression, and metastasis of cancer (138). The suppression of the immune system by MSCs is conducive to immune escape by tumour cells. Although MSCs have tumour homing properties, they can be used as vectors for antitumor drug delivery (139). However, the overall effect of MSCs on tumours is to promote tumour growth more than to inhibit tumour growth (138, 140). In patients treated with stem cells, the patient spontaneously developed gliomas after four years (141). Although there are few reports of tumorigenesis, this remains an aspect of MSC therapy that cannot be ignored. Although MSCs are beneficial to the recovery of neurological function in patients with SCI, the mechanism of action of MSCs and the cellular mechanisms that prevent the recovery of neural circuits after SCI remain unclear (41). Future research needs to focus on understanding the SCI cellular mechanisms and MSC action for use in clinical practice.

The fate of transplanted cells depends primarily on the surrounding environment rather than on the properties of the cells themselves (142). Inflammatory factors and toxic substances in the SCI microenvironment are not conducive to the survival of MSCs. Dead MSCs release toxic substances that exacerbate SCI damage (143). The survival rate and long-term survival of MSCs in hostile environments remain unresolved issues (144). Gels and scaffolds based on stem cell therapy can deliver MSCs to the site of injury and release MSCs slowly and continuously to the site of injury (145–149). Tissue engineering strategies can reduce the number of times that MSCs are administered to the patient and reduce the mechanical damage at the site of puncture. Various types of scaffolds and gels of natural and synthetic biomaterials have been developed to mimic the stem cell microenvironment to maintain stem cell survival (146). The ideal biomaterial should have good biocompatibility and low immunogenicity while being biodegradable. When transplanted to the site of injury, the biomaterial should have ideal mechanical properties for cell adhesion and axon regeneration (150). MSCs with neurotrophic factor co-delivery strategies appear to be more effective therapeutic options (151). However, tissue engineering is currently being applied only in the rat SCI injury model, and its application to clinical practice will require further research. We consider that tissue engineering strategies will address the challenges of delivering MSCs for the treatment of SCI in the future.

## 6 Conclusion

The amplification of the inflammatory cascade after SCI often causes permanent harm to the patient. Toxic substances and inflammatory factors not only aggravate SCI, but also cause irreparable damage to the nervous system. The role of MSCs in the treatment of SCI is promising. MSCs achieve immunosuppression through direct contact with immune cells or paracrine release signalling molecules to reduce the inflammatory response at SCI. MSCs also release neurotrophic factors such as BDNF and β-NGF to promote axon regeneration. Furthermore, MSCs regulate signalling pathways to inhibit glial scarring. Obstruction of glial scarring facilitates axon regeneration. In addition, MSCs can release angiogenic factors such as VEGF, FGF, PDGF and IGF-1 to promote perispinal angiogenesis and reshape BSCB. MSCs have been shown to be beneficial in SCI mice for motor function recovery in preclinical studies. In clinical practice, MSCs have also been used in the treatment of patients with SCI. The ability of MSCs to improve ASIA sensory and motor scores and promote bladder function recovery and neurological symptom recovery in patients with SCI has been widely demonstrated. After treatment with MSCs, patients mild fever, gastrointestinal dysfunction, headache, and urinary tract infections have been reported, although these did not cause serious adverse reactions. Although MSCs can improve ASIA sensory and motor scores in patients, the effectiveness of MSC therapy reported in the literature is not sufficient to support the widespread clinical use of MSCs and needs to be further improved. In addition, there are many unanswered questions about MSCs in the treatment of SCI. The cell dose and frequency of doses required, the mechanism of action of MSCs, and the cellular processes that prevent neural circuit recovery after SCI remain unclear. Furthermore, the toxic environment associated with SCI is not conducive to MSC survival. Although further clinical studies are required to elucidate these points, the evidence currently available indicates that MSCs are likely to play a major role in the treatment of SCI in the future.

## Author contributions

All authors read and approved the final manuscript. YL wrote the initial manuscript. CF contributed new ideas. JS created the figures. RH, YH and HY revised the manuscript and approved the final version.
